# Production and use of encapsidated RNA mimics as positive control reagents for SARS-CoV-2 RT-qPCR diagnostics

**DOI:** 10.1016/j.jviromet.2021.114372

**Published:** 2022-02

**Authors:** Hadrien Peyret, Elisabetta Groppelli, David Clark, Nicholas Eckersley, Tim Planche, Julian Ma, George P. Lomonossoff

**Affiliations:** a Department of Biochemistry and Metabolism, John Innes Centre, Norwich Research Park, Colney, NR4 7UH, UK; b Institute for Infection and Immunity, St. George's University of London, Cranmer Terrace, Tooting, London, SW17 0RE, UK

**Keywords:** Virus-like particles, Cowpea mosaic virus, SARS-CoV-2, RT-qPCR, Multiplexing, Diagnosis, In-tube control, Pseudogenome

## Abstract

The current gold standard technique for SARS-CoV-2 diagnostics is hydrolysis probe-based RT-qPCR. Reliable testing requires reliable control reagents to monitor the efficiency of RNA extraction, reverse transcription and PCR amplification. Here we describe a custom RNA packaging system from the plant virus cowpea mosaic virus to produce virus-like particles that encapsidate specifically designed portions of the genome of SARS-CoV-2, the causative agent of COVID-19. These encapsidated mimics are highly stable particles which can be used either to spike patient swab samples for use as an in-tube extraction and reaction positive control in multiplex RT-qPCR, or alone as a side-by-side mock-positive control reagent. The selection of sequences in the packaged pseudogenomes ensures that these mimics are compatible with the most commonly used primer/probe combinations for SARS-CoV-2 diagnostics (including German Berlin Charité Hospital, American CDC, and Chinese CDC protocols). The plant transient expression system used to produce these encapsidated mimics is inherently low-cost, and sufficiently high-yielding that a single laboratory-scale preparation can provide enough positive control reagent for millions of tests.

## Highlights

• Novel control reagents for SARS-CoV-2 RT-qPCR diagnostics based on RNA packaged within virus-like particles.
• Specific packaging of SARS-CoV-2 - derived RNA in cowpea mosaic virus particles *in vivo* in the plant *Nicotiana benthamiana*.
• Two different types of controls (In-Tube and Side-By-Side) produced for different applications.
• Suitable as controls for nucleic acid extraction, reverse transcription, and polymerase chain reaction steps.
• A single bench-scale preparation yields enough purified VLP controls for tens of millions of test reactions.

## 1 Introduction

The ongoing pandemic of coronavirus disease 2019 (COVID-19) is caused by the coronavirus SARS-CoV-2, and reverse transcriptase quantitative PCR (RT-qPCR) has been the main technique used to diagnose infections since the start of the pandemic (Corman et al., 2020). This involves nucleic acid extraction from a patient sample (usually a nasopharyngeal swab in universal transport medium (UTM) or viral transport medium (VTM)), followed by amplification and detection of the extracted nucleic acid by RT-qPCR. Even if partially automatable, the process involves multiple steps that carry inevitable risks of error or process failure that could compromise the validity of the result. Despite RT-qPCR being a highly refined diagnostic tool (and an exceptionally sensitive technique), its robustness, especially over thousands of samples as required by the COVID-19 pandemic, can be reduced by the occurrence of false negatives (Watson et al., 2020; Woloshin et al., 2020; Arevalo-Rodriguez et al., 2020). Therefore, it is crucial that reliable positive and negative controls validate the different steps in the process and increase the reliability of RT-qPCR diagnostic testing (Tahamtan and Ardebili, 2020).

Suitable positive controls for RT-qPCR consist of relatively short nucleic acids with known sequences that match the diagnostic primer and hydrolysis probe combinations. Although naked RNA sequences can be used as a reaction control in the RT-qPCR reaction, they are unsuitable as control for the entire diagnostic process as they are highly susceptible to degradation by RNases. Naked DNA is less labile but cannot control for the reverse transcription stage of the diagnostic test. Neither naked RNA nor DNA can be used as a control for the nucleic acid extraction step involved in SARS-CoV-2 infection diagnosis. Based on the available information from “instructions for use” leaflets of commercially-available kits for RT-qPCR detection of SARS-CoV-2, it appears that most manufacturers use a combination of naked DNA or naked RNA as their control reagents (see for example the COVID-19 RT-qPCR diagnostic kits from BioMerieux’s Argene® system, Genesig®, Krishgen BioSystems, and Kylt®). Some (such as Qiagen’s QIAstat-Dx® Respiratory SARS-CoV-2 Panel, Perkin Elmer®’s 2019-nCoV-PCR-AUS kit, and the Roche cobas® system) use armored RNA based on a non-specific RNA packaged inside MS2 bacteriophage particles (Pasloske et al., 1998). Meanwhile, SeraCare commercialises SARS-CoV-2 positive control reagents (Accuplex™) which are composed of recombinant alphavirus containing segments of the SARS-CoV-2 genome.

This manuscript describes a method for the production of RNA-based positive control reagents for SARS-CoV-2 diagnostic tests that relies on virus-like particle (VLP) technology. VLPs are nanoparticulate structures composed of viral structural proteins similar in structure to a native virion but which do not contain the infectious viral genome (Steele et al., 2017). Particles based on the non-enveloped plant virus cowpea mosaic virus (CPMV), a bipartite single-stranded positive-sense RNA virus (Order Picornavirales), can be readily produced and purified (Kruse et al., 2019). CPMV particles derived from genetically modified infectious virus have been used as diagnostic RT-qPCR control reagents for a virus of high economic importance, foot and mouth disease virus (FMDV, Madi et al., 2015; King et al., 2007). Our recent work has improved our understanding of the CPMV RNA packaging process, such that we are now able to produce non-infectious VLPs which package any RNA sequence of choice up to at least 6 kb in size (Kruse et al., 2019). This is done by inserting a synthetic sequence between the 5′ and 3′ untranslated regions (UTRs) of the CPMV RNA-2 genomic component, and this RNA construct is automatically packaged by CPMV coat proteins supplied in *trans* in the presence of the CPMV RNA-1 – encoded viral replication machinery *in vivo* in *Nicotiana benthamiana* plants. By using this technique, the specificity of CPMV RNA packaging is harnessed and allows selective packaging of custom RNA sequences in non-infectious particles. Therefore, we designed an RNA molecule that contains regions of the SARS-CoV-2 genome that are relevant for diagnostic purposes, which we term a pseudogenome, and generated CPMV VLPs that carry this RNA. We have further advanced the CPMV VLP production pipeline in plants to obtain the high-yielding packaging system described here (Peyret et al., 2019).

## 2 Results

### 2.1 Design of the encapsidated sequences

**Fig. 1:**
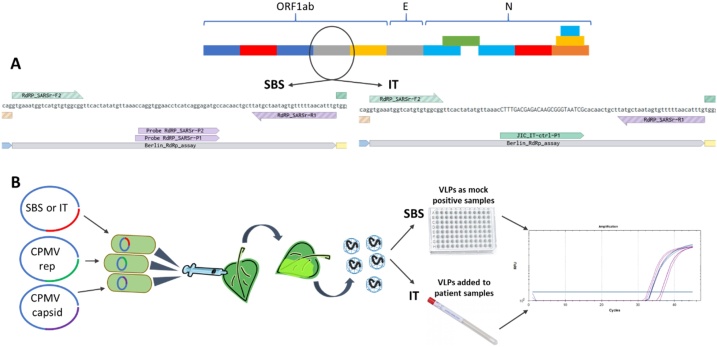
**The encapsidated mimic principle**. A): Schematic representation of the 1.2 kb pseudogenome containing the diagnostic target sequences from protocols listed in Table 1. Each box represents an amplicon colour coded by country of assay origin (dark blue: France; red: China; grey: Germany; gold: Hong Kong; cyan: USA; green: Thailand; orange: Japan), from three different regions of the SARS-CoV-2 genome (ORF1ab, E and N genes), some of which overlap. The difference between the side-by-side (SBS) and in-tube (IT) pseudogenomes are shown for one of the amplicons: the sequences are identical apart from the probe binding site, which allows differential detection of SBS from IT (and IT from wt virus) by multiplexing. Sequence diagrams obtained using Benchling. B): Expression plasmids encoding either the SBS or IT pseudogenome, the replication functions of CPMV (RNA-1), and the capsid precursor of CPMV (VP60) are transformed into *Agrobacterium tumefaciens* and co-expressed transiently in *Nicotiana benthamiana* plants. This results in *in vivo* packaging of the RNA pseudogenomes inside virus-like particles. After extraction and purification, these encapsidated mimics can be used as side-by-side (SBS) mock-positive controls, or as internal in-tube (IT) controls once added to a patient swab sample.

To meet the requirements of different diagnostic systems, two distinct encapsidated mimics are described here: a side-by-side (SBS) mock-positive reaction control and an in-tube (IT) extraction and reaction positive control (Fig. 1). Both are CPMV VLPs that contain a synthetic 1.2 kb RNA pseudogenome construct designed based on the reference SARS-CoV-2 genome (NCBI Reference Sequence: NC_045512.2). This construct includes all of the primer binding sites and amplicons of the seven RT-qPCR diagnostic test protocols that were described as technical guidance on the World Health Organisation (WHO) website on 18^th^ March, 2020 (see Table 1).

**Table 1:** Summary of SARS-CoV-2 genome targets included in encapsidated mimic pseudogenomes.

Country	Institute	Gene targets
China	China CDC	ORF1ab and N
Germany	Berlin Charité, Inst. Virology	RdRp, E, N
Hong Kong SAR	Hong Kong University	ORF1b-nsp14, N
Japan	National Institute of Infectious Diseases, Department of Virology	N
Thailand	National Institute of Health	N
USA	USA CDC	Three targets in N gene
France	Institut Pasteur, Paris	Two targets in RdRp

The SBS pseudogenome has the native sequence of the primer binding sites and amplicons, including probe binding sites. The SBS encapsidated mimic is therefore a mimic for wild-type SARS-CoV-2 and can be used as a non-infectious, non-hazardous control for use alongside patient samples in nucleic acid extraction and RT-qPCR diagnostics. By contrast, the IT pseudogenome includes the same primer binding sites and amplicons, but the probe binding sites have been replaced with different, novel synthetic sequences of the same length and melting temperature. The IT encapsidated mimic, therefore, is intended as an in-tube control to be added directly to a patient swab sample before nucleic acid extraction and RT-qPCR. Due to the modified probe binding site sequences, an additional control probe is added to the RT-qPCR reaction (labelled with a different fluorophore than the diagnostic probe) and the IT control can then be detected on a different fluorescence channel than the SARS-CoV-2 in a multiplex reaction. The sequences of the SBS and IT pseudogenomes can be found in Supplementary Fig. S1.

### 2.2 Production of SBS and IT encapsidated mimics

**Fig. 2:**
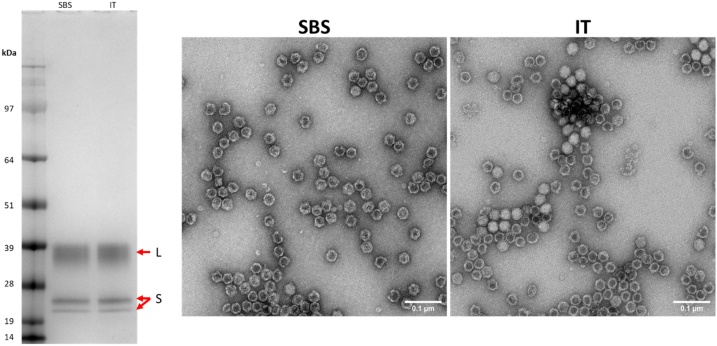
**SDS-PAGE and TEM analysis of purified preparations of SBS and IT encapsidated mimics**. Left: SDS-PAGE gel. The visible 39 and 23 kDa bands correspond to the large (L) and two electrophoretic forms of the small (S) coat proteins of CPMV, respectively, which make up the VLP capsid. Right: Transmission electron micrographs of SBS and IT VLPs after purification. Staining with 2% uranyl acetate, scale bars are 100 nm.

The SBS and IT pseudogenome sequences are flanked by the native 5’- and 3’-untranslated regions (UTR) of the CPMV RNA-2 genomic component. This allows the pseudogenomes to be packaged inside CPMV VLPs through replication-dependent packaging *in vivo* using *Agrobacterium tumefaciens* - based transient expression in *N. benthamiana* plants (Kruse et al., 2019). Each pseudogenome was transcribed in the plant leaves alongside a non-replicating construct which directs overexpression of the CPMV capsid protein precursor (VP60), and the CPMV RNA-1 genomic component which supplies the viral replication machinery and coat protein processing functions. The resulting VLPs were extracted from the plant leaves, purified, and analysed by SDS-PAGE (Fig. 2), which shows the 39 kDa Large and both electrophoretic forms of the ∼20 kDa Small coat proteins (Saunders et al., 2009; Montague et al., 2011). Negative stain transmission electron microscopy (Fig. 2) revealed typical CPMV VLPs, most of which appeared to take up the uranyl acetate stain, which is expected as most of the particles are RNA-free or contain a pseudogenome RNA which is significantly smaller than either of the CPMV genomic RNAs. Protein quantification of the preparations showed purified yields ranging from 0.2 to 1 mg of purified VLP per gram of fresh-weight infiltrated leaf tissue, which is the expected yield for CPMV VLPs (Sainsbury et al., 2014).

### 2.3 Thermal stability of encapsidated mimics

**Fig. 3:**
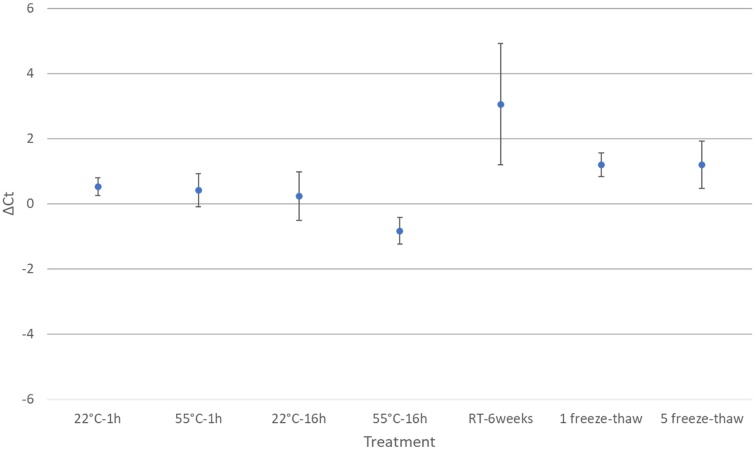
**Thermal stability of packaged pseudogenomes**. Aliquots of four separate preparations of CPMV VLP mimics were incubated at various temperatures for various lengths of time or submitted to freeze-thaw cycles as indicated before nucleic acid extraction and RT-qPCR. The graph shows the average difference in Ct value (ΔCt) between treated aliquots and control aliquots kept at 4 °C. RT: room temperature. Error bars represent 95 % confidence intervals.

Previously published work has shown that CPMV-based particles are stable at a broad range of temperatures that are likely to be encountered during production, transport and storage (King et al., 2007; Madi et al., 2015). To verify that this is also the case with these encapsidated mimics, four separate 100 ng preparations of encapsidated mimics (two SBS and two IT preparations) at 1 ng/μl in phosphate buffered saline (PBS) were subjected to various temperature conditions. These were: 22 °C for 1 h, 22 °C for 16 h, 55 °C for 1 h, 55 °C for 16 h, room temperature for 6 weeks, exposure to one freeze-thaw cycle or exposure to five freeze-thaw cycles. Nucleic acid was extracted from all samples and followed by RT-qPCR. The average Ct values for each treated sample was then compared to the average Ct value for the refrigerated reference aliquot for that sample (ΔCt). The results, shown in Fig. 3, indicate that these incubations and freeze-thawing of the encapsidated mimics had little effect on the final Ct value, indicating that the packaged pseudogenomes are stable over a broad range of conditions.

### 2.4 Effect of nucleic acid extraction on the encapsidated mimics

**Fig. 4:**
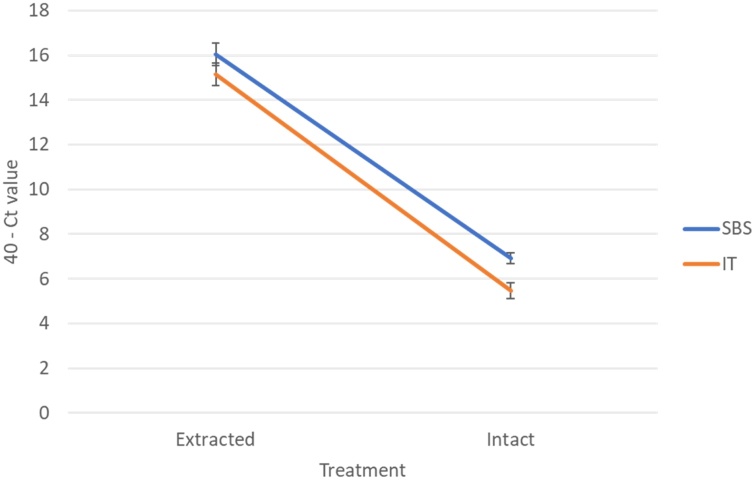
**Encapsidated mimics control for nucleic acid extraction**. SBS and IT VLPs at a final concentration of 0.5 ng/μl each were added to a swab sample before nucleic acid extraction (Extracted) or to elution buffer after nucleic acid extraction (Intact). RT-qPCR shows a difference of 10 cycles in detection of the packaged pseudogenomes, indicating that nucleic acid extraction leads to a thousand-fold improvement in detection of the pseudogenomes from the VLPs. The measured Ct are shown here as subtracted from 40 to show a more intuitive graphical representation of reduction in detectability. Error bars indicate standard deviation from three technical replicates.

To assess the extent to which the VLP capsid protects the RNA pseudogenome from enzymatic activity, a throat and nose swab from an individual who had tested negative for SARS-CoV-2 was divided in two, with one aliquot immediately spiked with both IT and SBS VLPs (125 ng of each for a final concentration of 0.5 ng/μl of each VLP). Both aliquots then underwent nucleic acid extraction, and the eluted nucleic acid from the unspiked aliquot was then spiked with SBS and IT VLPs (for a final concentration of 0.5 ng/μl of each). This resulted in two eluted nucleic acid samples from the same initial swab, but one sample contained pseudogenomes from VLPs that had undergone nucleic acid extraction (Extracted) while the other contained equivalent amounts of VLPs that had not undergone nucleic acid extraction (Intact). Both samples (Extracted and Intact) were then used as template in RT-qPCR, where the two different pseudogenomes were detected in the same tubes by multiplexing with different fluorophores. RNA from intact, non-extracted VLPs was detected about 10 cycles later than RNA from extracted VLPs (Fig. 4). This corresponds to a thousand-fold difference in detectable RNA, which may suggest that the CPMV capsid prevents access of reverse transcriptase to the pseudogenome; thus, nucleic acid extraction is necessary for efficient detection of the pseudogenomes. This is not surprising given that reverse transcription occurs at 55 °C for 10 min, and the data shown in Fig. 3 suggests that the encapsidated mimics are stable at this temperature for longer than this. This confirms that the encapsidated mimics can function as controls for the nucleic acid extraction step that precedes a SARS-CoV-2 diagnostic RT-qPCR.

### 2.5 Effect of the IT encapsidated mimic on patient samples and dilution effects

Excess Diagnostic Material (EDM) from patient nasopharyngeal swabs collected at St. George’s University Hospital (London, United Kingdom) was used to assess the utility of the IT encapsidated mimic as an in-tube control reagent. Five EDM samples were used, two of which (EDM 1 and 5) had been diagnosed positive for SARS-CoV-2 and three of which (EDM 8, 9 and 10) had been diagnosed negative. The EDM samples were then each split into two aliquots, one of which was spiked with 50 ng of IT VLP (for a final concentration of 1 ng/μl, for consistency with previous experiments). RNA was then extracted from all aliquots then used as template for two sets of RT-qPCR reactions using the RdRp and E assays described by Corman et al. (2020), with an extra probe added to each master mix to enable detection of the IT pseudogenome (IT probe conjugated to Cy5, SARS-CoV2 diagnostic probes conjugated to FAM). In all samples that contained the IT pseudogenome, this was consistently detected: at 24.3 (±0.1) Ct in the RdRp protocol and 21.2 (±0.1) Ct in the E protocol. Moreover, addition of the IT VLPs had no effect on diagnostic result of the negative EDM samples (no detection of SARS-CoV-2 below Ct of 37 in either RdRp or E protocol). However, for the two positive EDM samples, the presence of the IT control had a negative impact on detection of SARS-CoV-2 (Table 2). In EDM 1, SARS-CoV-2 was not detected when IT was present, but it was detected by both the RdRp and E protocol in the absence of IT at 32.3 and 28.4 Ct, respectively. In EDM 5, meanwhile, SARS-CoV-2 was detected when IT was present (at 30.7 and 31.9 Ct in the RdRp and E protocol respectively), but in both protocols this detection was at a higher Ct than in the absence of IT (28.8 and 25.4 Ct, respectively).

**Table 2:** Use of IT VLP in SARS-CoV-2 – positive Excess Diagnostic Material. Duplicate aliquots were prepared from two EDM samples which were positive for SARS-CoV-2. One aliquot for each was spiked with 50 ng IT VLP and all aliquots were subsequently used for RNA extraction and RT-qPCR using the Corman et al. (2020) RdRp and E protocols modified to allow multiplex detection of SARS-CoV-2 and the IT pseudogenome. The table shows the detection of SARS-CoV-2 (Patient) and IT pseudogenome (IT) in the unspiked (-IT) *versus* spiked (+IT) aliquots for both EDM samples (EDM 1 and EDM 5) as measured using the RdRp (left) and E (right) protocols. Values are average Ct from technical duplicate measurements.

		RdRp		E	
		-IT	+IT	-IT	+IT
EDM 1	Patient	32.3	not detected	28.4	not detected
	IT	not detected	24.3	not detected	21.1
EDM 5	Patient	28.8	30.7	25.4	31.9
	IT	not detected	24.5	not detected	21.2

These results indicate that in the positive EDM samples, the IT pseudogenome was orders of magnitude more abundant than SARS-CoV-2 RNA. These results suggest there is competition for primers between SARS-CoV-2 genomic RNA and the IT pseudogenome RNA, resulting in the signal for SARS-CoV-2 being outcompeted. To test this hypothesis, SBS encapsidated mimics were used as mock SARS-CoV-2 in mixes of SBS and IT VLPs at various ratios. RNA was extracted from 200 μL VLP mixes (in which the more abundant VLP was always at 0.5 ng/μl as measured by protein content) and this RNA was used as template for RT-qPCR using the Corman et al. (2020) RdRp protocol with an extra probe added to detect the IT pseudogenome. The results, shown in Table 3, reveal that the two pseudogenomes do compete in RT-qPCR reactions: while 1:1 or 10:1 ratios are well tolerated, 1000:1 ratios result in no detection of the less abundant pseudogenome after 40 PCR cycles.

**Table 3:** CPMV VLP-based mimics compete for primers at extreme ratios. Preparations of SBS and IT VLPs were mixed in various ratios followed by nucleic acid extraction and RT-qPCR. While the less abundant pseudogenome could still be detected in a ten-fold ratio, this was not the case for a thousand-fold ratio. Note that equal amounts of each VLP type were used based on protein quantification of the two samples, but RT-qPCR of each sample alone indicates that the SBS pseudogenome is more abundant than the IT pseudogenome. Values are Ct ± standard deviation from three technical replicates.

	Ct	
SBS:IT ratio	SBS	IT
each alone	24.35 ± 0.37	26.1 ± 0.04
1:1	24.29 ± 0.13	25.3 ± 0.1
10:1	24.35 ± 0.07	28.15 ± 0.1
1:10	26.86 ± 0.17	25.33 ± 0.14
1000:1	24.23 ± 0.18	not detected
1:1000	not detected	25.8 ± 0.28

In this experiment, 100 ng (as measured by protein content) of VLP in a 200 μL mix was always used as starting material for the more abundant VLP (*i.e.* the 1:1 ratio was a mix of 100 ng of each VLP and the 1:1000 ratio was a mix of 0.1 ng of SBS and 100 ng of IT VLP). After RNA extraction, 5 % of the eluted RNA was used in each RT-qPCR reaction (2.5 μL of 50 μL eluate), which corresponds to RNA from 5 ng of VLP for the more abundant VLP. Similarly, in the experiments with spiked EDM (Table 3), 50 ng of IT VLP (from a 5 mg/ml stock) were added to EDM aliquots before RNA extraction and 5.7 % of the eluted RNA was used in each RT-qPCR reaction (2 μL of 35 μL eluate), which corresponds to RNA from approximately 2.9 ng of VLP. The relatively low Ct values indicate that this is far more than is necessary for detection of the encapsidated mimics.

By comparing RNA extracted from a range of concentrations of SBS encapsidated mimics with a standard curve of *in vitro* transcripts of SARS-CoV-2 RNA, we calculated that the pseudogenome content of the VLPs is in the range of hundreds of thousands of pseudogenome copies per ng of VLP: 3.59 × 10^5^ ± 7.75 × 10^3^ copies per ng of VLP as measured using the RdRp protocol, and 1.82 × 10^5^ ± 7.38 × 10^3^ copies per ng of VLP as measured using the E protocol from Corman et al. (2020).

## 3 Discussion

Here we describe two types of positive control reagents for use in RT-qPCR – based SARS-CoV-2 diagnostic testing. Both types of reagents are encapsidated mimics: VLPs made in plants which contain synthetically designed RNA pseudogenomes that correspond to segments of the SARS-CoV-2 genome that are typically used as diagnostic amplicons. Both types of encapsidated mimics are readily produced at high yields, are thermostable, and provide protection for the RNA from degradation and enzymatic activity (or at least reverse transcription activity) until after RNA extraction. This means that both types of encapsidated mimics are suitable for use as controls for the RNA extraction process. Moreover, a serial dilution experiment on four separate preparations of encapsidated mimics revealed that using 1 ng of VLP for RNA extraction, then 5 % of the eluate (corresponding to RNA from 0.05 ng of VLP) in each RT-qPCR reaction consistently allowed detection at 35 Ct (± 1.7 Ct) with the RdRp protocol from Corman et al. (2020). This means that 1 ng of VLP added to a patient swab sample (for the IT mimic) or to a control sample (for the SBS mimic) before RNA extraction is sufficient for consistent detection of the control pseudogenome. Given the purified yield of these VLPs in the plant transient expression system (up to 1 mg of VLP per gram of infiltrated leaf tissue), this means that one gram of infiltrated leaf tissue can yield enough SBS encapsidated mimic for about one million batches of tests, or enough IT encapsidated mimic to add to approximately 1 million patient swab samples. A standard laboratory-scale preparation (60−70 g of infiltrated leaf material) could therefore yield enough encapsidated mimics to test the entire population of the United Kingdom. Along with large quantities, plants can provide a reliable and consistent source of these reagents thanks to the exceptional stability of the CPMV-based particles and the existing industrial capacity for producing biologics in plants (Montague et al., 2011; Fischer and Buyel, 2020).

It should be noted that the majority of the VLPs produced by transient expression do not contain pseudogenome RNA but instead contain either CPMV RNA-1 or are devoid of any RNA. While the ratios of each of the subtypes of VLPs is consistent from batch to batch, this ratio can be heavily affected by the specific purification procedure used. For example, some techniques such as solvent extraction steps can disproportionately destabilise (and ultimately remove) RNA-free VLPs compared to RNA-containing VLPs. Any large-scale or commercial deployment of these VLPs (which may use a different purification procedure as the one described here) may therefore result in a different pseudogenome-to-VLP protein ratio, and this would need to be assessed. If required, it is possible to separate CPMV VLPs based on their RNA content by density gradient ultracentrifugation (typically done in caesium chloride), resulting in pure pseudogenome-containing VLPs. This was not done for the current study and is not expected to be necessary for diagnostic applications.

The IT mimic is an internal control intended to provide confidence that a negative RT-qPCR result in a patient sample is valid: *i.e.* that the nucleic acid extraction and RT-qPCR reactions took place successfully. The American CDC recommends detecting human RNase P in swab samples alongside SARS-CoV-2 as a swab quality control, but the use of the IT control offers complementary information. Indeed, while the RNase P RT-qPCR assay controls for the swab having been taken and transported properly, it does not control for the reverse transcription reaction or RNA stability, because the recommended primer/probe combination binds entirely to exon 1 of the human RNase P gene (GenBank entry NM_006413.5), and so will amplify genomic DNA. The IT VLP cannot control for the quality of the swab, but it can control for its correct processing, RNA extraction, reverse transcription, and quality of the specific diagnostic primers, since these amplify both viral RNA and the IT pseudogenome. However, the data presented here show that this will only be the case if care is taken to use a small enough amount of IT mimic in each patient swab sample to ensure that any SARS-CoV-2 viral RNA will not be outcompeted by excess IT control pseudogenome, which is amplified using the same primers as SARS-CoV-2 targets during reverse transcription and PCR. Nevertheless, our data indicate that there is a tolerance for a 10-fold excess of one target (viral or control) compared to the other. Our data therefore support the recommendation that if the IT mimic is used, it should be in an amount where signal from the IT pseudogenome is expected at about 35 Ct or higher (which corresponds to using the amount of RNA extracted from about 0.05 ng of IT VLP in the RT-qPCR reaction). This will minimise primer sequestration and enable SARS-CoV-2 detection at a high sensitivity (many diagnostic tests will use a cut-off Ct of 35–37 for determining whether a sample is positive or negative for SARS-CoV-2).

The SBS mimic is a more standard type of control that is run alongside (and not within) patient samples, and so can also be used for virus quantification rather than purely Ct results. Unlike the IT mimic, this control can be used as a mock patient sample at a wide range of concentrations, but RNA from 0.05 to 1 ng of SBS VLP in an RT-qPCR reaction is expected to yield a clear, consistent signal. In any event, differences in the characteristics of the patient swab samples, RNA extraction protocols, and RT-qPCR protocols will require situational optimisation of the use of SBS and IT encapsidated mimics. The data presented here supports carrying out these optimisations first and foremost with a range of 0.1−10 ng of VLP added to real or mock patient swab samples. The availability of effective, stable, non-infectious mimics of SARS-CoV-2 should also enable effective proficiency testing enabling the comparison of data obtained using different PCR protocols and machines. The SBS encapsidated mimic is already being deployed as a positive control for large-scale SARS-CoV-2 diagnostic testing by the Norwich Testing Initiative based at the Earlham Institute, Norwich, UK, for large-scale diagnostic testing of a university population (Berger Gillam et al., 2021).

None of the amplicons present in the pseudogenome originated from the Spike (S) gene of SARS-CoV-2, and this pseudogenome is, to our knowledge, still valid against the variants of concern (VOC) that have evolved since the beginning of the pandemic. However, it is possible to develop variant-specific encapsidated mimics by modifying the packaged pseudogenome, and this is not expected to have a detectable effect on the yield or physical/chemical characteristics of the VLPs. Similarly, it is important to note that this technology is not inherently specific to SARS-CoV-2, and can readily be applied to create bespoke VLP-based RT-qPCR control reagents for any RNA virus for which genome sequence information is available.

## 4 Materials and methods

### 4.1 Generation of the encapsidated mimics

The World Health Organisation (WHO) website accessed on the 18^th^ of March, 2020 (https://www.who.int/emergencies/diseases/novel-coronavirus-2019/technical-guidance/laboratory-guidance) was used to identify the primer and probe binding sites for the RT-qPCR – based SARS-CoV-2 diagnostic tests available at the time. These probe and primer sequences were used to identify the corresponding amplicons in the reference SARS-CoV-2 genome (NCBI Reference Sequence: NC_045512.2), and this allowed the design of the SBS and IT pseudogenomes (sequences available in the Supplementary Information), which were ordered for DNA gene synthesis from GeneArt (ThermoFisher Scientific). The pseudogenomes were then cloned between the wild-type sequences of the CPMV RNA-2 5’- and 3’- untranslated regions (UTRs) in the pEAQ-GFP expression vector (Kruse et al., 2019) to yield pEAQ-SARS-CoV-2-SBS and pEAQ-SARS-CoV-2-IT, containing the SBS and IT pseudogenomes, respectively. In parallel, the VP60 gene of CPMV, which encodes the capsid protein precursor of CPMV, was taken from pEAQ-*HT*-VP60 (Saunders et al., 2009) and cloned into the pHREAC overexpression vector (Peyret et al., 2019) to yield pHREAC-VP60. All plasmids were transformed into chemically competent *E. coli* TOP10 cells (Invitrogen) and plasmid DNA from transformed colonies was verified by gene sequencing (Eurofins Scientific) before transformation into electrocompetent *Agrobacterium tumefaciens* LBA4404. These were then used for transient expression in *Nicotiana benthamiana* plants *via* agroinfiltration using a needleless syringe as described previously (Meshcheriakova et al., 2014). Co-infiltration was used to co-express the pseudogenome-encoding pEAQ vectors alongside both pHREAC-VP60 and pEAQ-RNA-1-Int (Kruse et al., 2019), which encodes the RNA-1 genomic component of CPMV. Plants were grown in greenhouses maintained at 25 °C with supplemental lighting to provide 16 h of daylight.

### 4.2 Encapsidated mimic extraction, purification and characterisation

Infiltrated leaves were harvested seven days post-infiltration (dpi), and particles were purified from these by polyethylene glycol (PEG) precipitation and pelleting as described previously (Sainsbury et al., 2014). Following pelleting by ultracentrifugation, VLPs were resuspended overnight on a cold room shaker in 10 mM sodium phosphate pH 7.2. They were then clarified by two successive rounds of centrifugation at 16,000 × g for 20 min at 8 °C. Micrococcal nuclease digests (New England Biolabs) were then carried out at 37 °C for 20 min to digest any unencapsidated nucleic acid that may have carried over during the purification process. This was followed by the addition of EGTA to a final concentration of 10 mM, followed by filtration through a Minisart 0.2 μm syringe filter (Sartorius) into an Amicon spin concentrator with a 100 kDa molecular-weight cut-off. This was used for buffer-exchange into fresh 10 mM sodium phosphate to remove nuclease and EGTA. Samples were then clarified again at 16,000 × g for 20 min at 8 °C. Finally, sodium azide was added to each sample at a final concentration of 3 mM for storage in the refrigerator. VLP preparations were quantified by protein content using a bicinchoninic acid (BCA) protein assay kit (Pierce) with a bovine serum albumin standard curve. The protein content of the preparations was then visualised by electrophoresis of denatured samples on 12 % (w/v) Bis-Tris NuPAGE gels (Life Technologies) after boiling 2 μg of the samples in Invitrogen NuPAGE LDS sample buffer and staining of the SDS-PAGE gel with InstantBlue (Expedeon).

### 4.3 RNA extraction and RT-qPCR

Nucleic acid extraction was carried out using the GeneJET Viral DNA/RNA Purification Kit (ThermoFisher Scientific) as per manufacturer’s instructions, except for the EDM spiking experiments, for which nucleic acid extraction was carried out using the QIAamp Viral RNA Mini Kit (Qiagen). RT-qPCR was done according to the protocol described by Corman et al. (2020) for the RdRp and E targets using the SuperScript III One-Step RT-PCR System with Platinum Taq DNA Polymerase (ThermoFisher Scientific). For the RdRp protocol, primers RdRp_SARSr-F (5’- GTGARATGGTCATGTGTGGCGG – 3’) and RdRp_SARSr-R (5’- CARATGTTAAASACACTATTAGCATA -3’), and probe RdRp_SARSr-P2 (5’- FAM-CAGGTGGAACCTCATCAGGAGATGC-BBQ -3’) along with the control probe to detect the IT pseudogenome (JIC_IT-ctrl-P1: 5’- Cy5-CTTTGACGAGACAAGCGGGTAATCG-BHQ2 -3’) were custom synthesised by Eurofins Scientific. For the E protocol, primers E_Sarbeco_F (5’- ACAGGTACGTTAATAGTTAATAGCGT -3’) and E_Sarbeco_R (5’- ATATTGCAGCAGTACGCACACA -3’), and probe E_Sarbeco_P1 (5’- FAM-ACACTAGCCATCCTTACTGCGCTTCG-BHQ1 -3’) along with the control probe to detect the IT pseudogenome (JIC_IT-GER-E-P1: 5’- Cy5- GAACCGCCGCCATAGTTAGCTTCAAC-BHQ2 -3’) were custom synthesised by Sigma Aldrich (Merck). The IT control probes were both used at a concentration of 200 nM per reaction, and all other oligonucleotides were used in the concentrations recommended by Corman et al. (2020). All RT-qPCR reactions were carried out using BioRad CFX96 Touch Real-Time PCR thermocyclers using the FAM and Cy5 channels.

## Authors contributions

HP designed constructs and HP and GPL conducted expression tests in plants and purification and analysis of VLPs. HP carried out experiments that did not involve EDM. EG, DC, NE and TP carried out the experiments with EDM. JM provided critical assistance coordinating work between JIC and SGUL. All authors contributed to the manuscript.

## Author statement

HP, GPL: Conceptualization; HP, EG, DC, NE: Data curation; All authors: Formal analysis; JM, GPL: Funding acquisition; HP, EG, DC, NE: Investigation; All authors: Methodology; HP, JM: Project administration; HP, GPL, JM: Resources; EG, DC, TP, GPL: Supervision; HP: Writing - original draft; All authors: Writing - review & editing.

## Data availability

The data are presented in the figures and in the Supplementary material.

## Declaration of Competing Interest

The authors declare that they have no known competing financial interests or personal relationships that could have appeared to influence the work reported in this paper.
