# Supplementary material for: Production and use of encapsidated RNA mimics as positive control reagents for SARS-CoV-2 RT-qPCR diagnostics
Source: J Virol Methods. 2022 Feb;300:114372. doi: 10.1016/j.jviromet.2021.114372 (PMC8611828; doi:10.1016/j.jviromet.2021.114372)
Supplement: Supplementary file 1 [file mmc1.docx]

Supplementary Material

**Production and use of encapsidated RNA mimics as positive control reagents for SARS-CoV-2 RT-qPCR diagnostics.**

Hadrien Peyret*^1^, Elisabetta Groppelli^2^, David Clark^2^, Nicholas Eckersley^2^, Tim Planche^2^, Julian Ma^2^, George P. Lomonossoff^1^

1: Department of Biochemistry and Metabolism, John Innes Centre, Norwich Research Park, Colney NR4 7UH, UK

2: Institute for Infection and Immunity, St. George's University of London, Cranmer Terrace, Tooting, London SW17 0RE, UK

* Corresponding author: [Hadrien.peyret@jic.ac.uk](mailto:Hadrien.peyret@jic.ac.uk)

**Supplementary Figure S1: RNA sequences of the encapsidated mimic pseudogenomes.** Sequences highlighted in yellow are the flanking regions of the pseudogenome (derived from the cowpea mosaic virus genome) necessary for its production. The probe binding sites (underlined) for each test are authentic in the Side-by-Side (SBS) pseudogenomes but have been replaced with new sequences in the In-Tube (IT) pseudogenome. These are detectable by multiplexing in the same reaction using the same primers as the diagnostic test but different hydrolysis probes (conjugated to different fluorophores). Note that the probe binding site for the Thai National Institute of Health test could not be changed in the IT pseudogenome because this probe binding site is contained within a primer binding site for the American CDC test. The IT encapsidated mimic is therefore not suitable for use as an in-tube control with the Thai National Institute of Health test.

**Sequence of the side-by-side (SBS) control pseudogenome:**

For us in probe-based RT-qPCR diagnostic tests:

- Add VLPs to patient sample before RNA extraction
- Detect using diagnostic primers (same amplification) but different probe
- Differentiate diagnostic probe (against virus) from control probe (against pseudogenome) thanks to different fluorophore (multiplexing)
- Control probe detection indicates successful extraction and amplification
- Dramatically reduces false negatives

uauuaaaaucuuaauagguuuugauaaaagcgaacguggggaaacccgaaccaaaccuucuucuaaacucucucucaucucucuuaaagcaaacuucucucuugucuuucuugcaugagcgaucuucaacguugucagaucgugcuucggcaccaguacaauguuuucuuucacugaagcgaaaucaaagaucucuuuguggacacguagugcggcgccauuaaauaacguguacuuguccuauucuugucgguguggucuugggaaaagaaagcuugcuggaggcugcuguucagccccauacauuacuuguuacgauucugcugacuuucggcgggugcaauaucucuacuucugcuugacgagguauuguugccuguacuucuuucuucuucuucuugcugauugguucuauaagaaaucuaguauuuucuuugaaacagaguuuucccgugguuuucgaacuuggagaaagauuguuaagcuucuguauauucugcccaaauucGACCGgaccgguuuaugagcuuaguccuguugcacuacgacagaugucuugugcugccgguacuacacaaacugcuugcacugaugacaaugcguuagcuuacuacaacacaacaaagggagcccuguggguuuuacacuuaaaaacacagucuguaccgucugcgguauguggaaagguuauggcuguaguugugaucaacuccgcgaacccaugcuucagucagcugaugcacaaucgugguaacugguaugauuucggugauuucauacaaaccacgccagguaguggaguuccuguuguagauucuuauuauucauuguuaaugccuauauuaaccuugaccaggugaaauggucauguguggcgguucacuauauguuaaaccagguggaaccucaucaggagaugccacaacugcuuaugcuaauaguguuuuuaacauuugugggguuuuacagguaaccuacaaagcaaccaugaucuguauugucaaguccaugguaaugcacauguagcuaguugugaugcaaucaugacuaggugucuagcuguccacgagugcuuuguuaagcguguucacgcggaucgcauugagaacccggaauaucgcgaaacagucgacuagguaacugacuagccuaggggugggucugguauuaauacagguacguuaauaguuaauagcguacuucuuuuucuugcuuucgugguauucuugcuaguuacacuagccauccuuacugcgcuucgauugugugcguacugcugcaauaugaccccaaaaucagcgaaaugcaccccgcauuacguuugguggacccucagauucaacuggcaguaaccagaauggagaacgcaguggggagccuugaauacaccaaaagaucacauuggcacccgcaauccugcuaacaaugcugcaaucgugcuacaggggaacuucuccugcuagaauggcuggcaauggcggugaugcugcucuugcuuugcugcugcuugacagauugaaccagcuugagagcaaaaugucugaaauuuuggggaccaggaacuaaucagacaaggaacugauuacaaacauuggccgcaaauugcacaauuugcccccagcgcuucagcguucuucggaaugucgcgcauuggcauggaagucacaccuucgggaacgugguugaccuacacaggugccacucgaggccuuuaacucugguuucauuaaauuuucuuuaguuugaauuuacuguuauucggugugcauuucuauguuuggugagcgguuuucugugcucagaguguguuuauuuuauguaauuuaauuucuuugugagcuccuguuuagcaggucgucccuucagcaaggacacaaaaagauuuuaauuuuauuaaaaaaaaaaaaaaaaaa

**Sequence of the in-tube (IT) control pseudogenome:**

For us in probe-based RT-qPCR diagnostic tests:

- Add VLPs to patient sample before RNA extraction
- Detect using diagnostic primers (same amplification) but different probe
- Differentiate diagnostic probe (against virus) from control probe (against pseudogenome) thanks to different fluorophore (multiplexing)
- Control probe detection indicates successful extraction and amplification
- Dramatically reduces false negatives

UauuaaaaucuuaauagguuuugauaaaagcgaacguggggaaacccgaaccaaaccuucuucuaaacucucucucaucucucuuaaagcaaacuucucucuugucuuucuugcaugagcgaucuucaacguugucagaucgugcuucggcaccaguacaauguuuucuuucacugaagcgaaaucaaagaucucuuuguggacacguagugcggcgccauuaaauaacguguacuuguccuauucuugucgguguggucuugggaaaagaaagcuugcuggaggcugcuguucagccccauacauuacuuguuacgauucugcugacuuucggcgggugcaauaucucuacuucugcuugacgagguauuguugccuguacuucuuucuucuucuucuugcugauugguucuauaagaaaucuaguauuuucuuugaaacagaguuuucccgugguuuucgaacuuggagaaagauuguuaagcuucuguauauucugcccaaauucGACCGgAccgguuuaugagcuuaguccuguugcacuacgacACAATACGCATCACGCCAGTCcuacacaaacugcuugcacugaugacaaugcguuagcuuacuacaacacaacaaagggagcccuguggguuuuacacuuaaaaacacagucuguaTCTTCTCACGTCCTGACGGAGTAGCTGCcuguaguugugaucaacuccgcgaacccaugcuucagucagcugaugcacaaucgugguaacugguaugauuucggugauuTGCTCTTGTAGGCGACTCGuaguggaguuccuguuguagauucuuauuauucauuguuaaugccuauauuaaccuugaccaggugaaauggucauguguggcgguucacuauauguuaaacCTTTGACGAGACAAGCGGGTAATCGcacaacugcuuaugcuaauaguguuuuuaacauuugugggguuuuacagguaaccuacaaagcaaccaugaucuguauugucaaguccaugguaaugcacauguagcATCTGGAATAGTTAACGTAGCTGAgugucuagcuguccacgagugcuuuguuaagcguguucacgcggaucgcauugagaacccggaauaucgcgaaacagucgacuagguaacugacuagccuaggggugggucugguauuaauacagguacguuaauaguuaauagcguacuucuuuuucuugcuuucgugguauucuugcuaguuGAACCGCCGCCATAGTTAGCTTCAACauugugugcguacugcugcaauaugaccccaaaaucagcgaaaugcGGTACGCTGACCGTTTGGTGGACCcucagauucaacuggcaguaaccagaauggagaacgcaguggggagccuugaauacaccaaaagTGCTAATGAGGGCGCTGCGGCTATcuaacaaugcugcaaucgugcuacaggggaacuucuccugcuagaauggcuggcaauggcggugaugcugcucuugcuAGAATGCGGATTGATTCACCgaaccagcuugagagcaaaaugucugaaauuuuggggaccaggaacuaaucagacaaggaacugauuacaaacauuggccgcaaaAAGGTGGATGGTCGTGGAGGGAGGCTTcguucuucggaaugucgcgcgcaaacauggaagucacaccuucgggaacgugguugaccuacacaggugccacuCGAGgccuuuaacucugguuucauuaaauuuucuuuaguuugaauuuacuguuauucggugugcauuucuauguuuggugagcgguuuucugugcucagaguguguuuauuuuauguaauuuaauuucuuugugagcuccuguuuagcaggucgucccuucagcaaggacacaaaaagauuuuaauuuuauuaaaaaaaaaaaaaaaaaa
